# Supplementary material for: The tadpole of Chiasmocleis altomontana (Anura: Microhylidae)
Source: PeerJ. 2025 Apr 16;13:e19220. doi: 10.7717/peerj.19220 (PMC12009030; doi:10.7717/peerj.19220)
Supplement: Supplemental Information 1 [file peerj-13-19220-s001.docx]

APPENDIX 1

Voucher specimens

*Chiasmocleis altomontana*, BRAZIL: São Paulo: São José do Barreiro: Parque Nacional da Serra da Bocaina, CFBH 28800-28806, DZSJRP-Amphibia-Adults 12147-12152; DZSJRP-Tadpoles L. 2066.02 (Base - PT3), 2036.02 (Trilha do Ouro – PT), 2035.01 (Flora Mariana), 2021.01 (Flora Mariana), 2046.02 (Base PT3), 2054.01 (Base PT2), 2097.01 (Base - PT3).
